# Not now but later – a qualitative study of non-exercising pregnant women’s views and experiences of exercise

**DOI:** 10.1186/s12884-018-2035-3

**Published:** 2018-10-11

**Authors:** Maria Ekelin, Mette Langeland Iversen, Mette Grønbæk Backhausen, Hanne Kristine Hegaard

**Affiliations:** 1 Department of Health Sciences, Lund University, PO Box 157, S-22100 Lund, Sweden; 2 Department of Obstetrics, Copenhagen University Hospital, Rigshospitalet, Copenhagen, Denmark; 3 The Research Unit Women’s and Children’s Health, The Juliane Marie Centre, Copenhagen University Hospital, Rigshospitalet, Blegdamsvej 9, 2100 Copenhagen, Denmark; 4 The Institute of Clinical Medicine, Faculty of Health and Medical Sciences, University of Copenhagen, Blegdamsvej 3, Copenhagen, Denmark; 5 Department of Gynecology and Obstetrics, Zealand University Hospital, Sygehusvej 10, 4000 Roskilde, Denmark

**Keywords:** Pregnancy, Exercise, Sedentary lifestyle, Experience, Health behaviour

## Abstract

**Background:**

Evidence has shown that there are several physical and mental advantages of exercise during pregnancy. Despite this, the recommendations for exercise during pregnancy are poorly fulfilled. The aim of this study was to illuminate non-exercising pregnant women’s views and experiences concerning exercise before and during pregnancy.

**Method:**

The study had a qualitative design with an inductive approach and was analysed by content analysis. A total of 16 individual and face-to-face interviews were conducted with healthy pregnant women, mainly in the third trimester and living in Sweden. The participating women had not been exercising 3 months before pregnancy or during pregnancy.

**Results:**

The main category “Insurmountable now, but possible in the future” was based on the four categories: “Lost and lack of routines”, “Feelings of inadequacy”, “Having a different focus” and “Need for support”. The women experienced that their lack of routines was a major barrier that prevented them from exercising. Other factors that contributed were, for example, pregnancy-related problems, long working days and prioritizing family life. The women described it as difficult to combine exercise with their focus on the pregnancy and they missed continuous support from the antenatal care provider. The women expressed a need for suggestions concerning exercise during pregnancy and follow-up on previous counselling, especially when pregnancy-related issues arose. Information about easily accessible alternatives or simple home exercises was requested. They felt immobile and were not satisfied with their inactivity and tried to partly compensate with everyday activities. The women identified the postpartum period as an important possibility for becoming more active, for their own sake, but also because they wanted to become role models for their children.

**Conclusion:**

Continuous support during pregnancy is needed concerning exercise. Pregnancy is mostly a barrier that prevents exercise for this group of women but, at the same time, may be a motivator and a possibility for better health. As the result showed that these women were highly motivated to a life-style change post-pregnancy, it may be crucial to support previously non-exercising women postpartum.

**Electronic supplementary material:**

The online version of this article (10.1186/s12884-018-2035-3) contains supplementary material, which is available to authorized users.

## Background

The focus on the importance of exercise during pregnancy has been consistent since the American Congress of Obstetricians and Gynecologists published the recommendation for exercise in 2002 [1]. Today ACOG recommends exercise of moderate intensity for at least 20–30 min on most or all days of the week during pregnancy and the postpartum period [2]. In the years since then, many countries have published similar recommendations [3]. Exercise has been defined as physical activity consisting of planned, structured and repetitive bodily movements done to improve and/or maintain one or more components of physical fitness [4]. However, recent studies have shown that recommendations for exercise during pregnancy are poorly fulfilled [5–9].

This is a concern as it is well documented that exercise during pregnancy is associated with a lower risk of adverse pregnancy outcomes such as gestational diabetes mellitus [10], preeclampsia [11], infants with excessive birth weight (> 4000 g) [7], low back pain [12] and caesarean section [13]. Exercise may also be effective in reducing symptoms of depression during pregnancy among healthy women and women at risk of depression [14, 15].

Pre-pregnancy exercise has shown to be a strong predictor for physical activity during pregnancy [5, 7, 16, 17] and women who exercise before pregnancy have been characterized by having a higher level of income and education, more often being married or living with a partner compared to non-exercising women [18]. Furthermore, lower education level, smoking, overweight, and not speaking the native language have been identified as predictors for not meeting the recommendations for exercise during pregnancy [5]. This may reflect a social inequality concerning exercise during pregnancy, which can lead to social differences in health [19].

In Sweden, midwives have the responsibility for the antenatal care of healthy women. In early pregnancy, the midwife has an informative dialogue with the woman concerning health and lifestyle [20].The Swedish recommendations [20] for exercise during pregnancy are in line with the international recommendations [2, 3] and it was recently shown in a large Swedish study population that 47% of the women met the recommendation for exercise in early pregnancy [21].

Non-exercising pregnant women seem to be particularly in need of advice and support from health professionals concerning exercise and healthy lifestyle. However, Hegaard et al. [17] showed that among women mainly having a sedentary level of leisure-time physical activity before pregnancy, only one in four increased their activity during pregnancy.

Previous quantitative and qualitative studies have explored the reasons why women who exercise before pregnancy reduced their engagement in exercise during pregnancy [22–24]. However, less is known concerning healthy pregnant women who do not engage in physical exercise both before and during pregnancy. More knowledge is needed in order to support exercise and health in these women during pregnancy and in the postpartum period.

### Aim

The aim of this study was to illuminate non-exercising pregnant women’s views and experiences concerning exercise before and during pregnancy.

## Method

This study had a qualitative design with an inductive approach.

### Participants

From January 2015 to June 2016 16 face-to-face interviews were performed with pregnant women. The inclusion criteria were Swedish- or English-speaking non-exercising women, and 18 years of age or older. For the purpose of this study non-exercising women were defined as those neither exercising in the last 3 months before pregnancy nor during pregnancy. Women with conditions that contra-indicated exercise were excluded, as well as women diagnosed with gestational diabetes, as they receive special information from the health professionals concerning physical activity.

In line with other studies, we exemplified exercise as swimming, training in water, dancing, yoga, jogging, ball games, fitness, cycling, horseback riding, and other types of similar exercise [5, 25]. The sampling strategy was purposeful sampling with consecutive recruitment. The participating women were enrolled at three different antenatal care centres in the south of Sweden, two situated in a larger city, and one in a rural area. Pregnant women were recruited by their midwife when undergoing a routine glucose tolerance test offered to all pregnant women around gestational week 28, or earlier if the woman has risk factors for diabetes, after having been given verbal and written information about the study. Women who agreed to participate were contacted by the researchers. Initially 21 women agreed to be contacted and 16 finally agreed to take part. Written informed consent was obtained from all participants.

### Data collection

One woman was interviewed in the first trimester, all the other women in the third trimester. The interviews took place where the participants preferred, two at the University of Lund and the remainder elsewhere: the antenatal care centre, a café, a library and at the woman’s home. The interviews were all conducted by the first author, individually and face to face. Each interview lasted approximately 30 min.

The interviews all started by asking the same overall question: “What are your views and experiences concerning exercise before and during pregnancy?” This was followed by clarifying questions, such as “What do you mean?” or “Can you explain further?”. Please see Additional file 1.

The interviews were audio taped and transcribed verbatim by the first author.

### Analysis

Data were analysed using content analysis [26, 27] as it is considered a suitable method widely used for developing and extending knowledge in health sciences by interpreting the meaning of text data [27, 28]. Firstly, the interviews were read through to get a sense of the whole. Then, open inductive coding was performed by writing notes consisting of short sentences or single words in the text margin, which summed up what was being said. The initial coding process started after the first interview. Throughout the whole process, memos were made about the topics and categorization of data. The codes, and the text sections belonging to them, were then read, re-read and compared. In this process similar or overlapping codes were grouped together into sub-categories. Subsequently the sub-categories were compared and merged, thereby reducing them in number. The sub-categories were finally sorted into categories until consensus was reached between the authors. Throughout the process of analysis, the transcripts were re-read to verify the emerging findings. All authors were involved in the process of analysis.

### Ethics and consent statement

Informed written consent was obtained prior to interviews. The study was approved by the Regional Research Ethics Board (Reg. no. 2014/733). All of the data were treated confidentially.

## Results

The women were 23–41 years old and eight were expecting their first child, while the remaining eight women were expecting their second or third baby. The characteristics of the women are presented in Table 1. One women was not Swedish-speaking and was therefore interviewed in English.

**Table 1:** Characteristics of the women

Age range:	23–41 years
Education:	
High school	6
University/college	10
Expecting child:	
First	8
Second	5
Third	3
Occupation:	
Working	11
Maternity leave	3
Sick leave	1
Unemployed	1

**Fig. 1:**
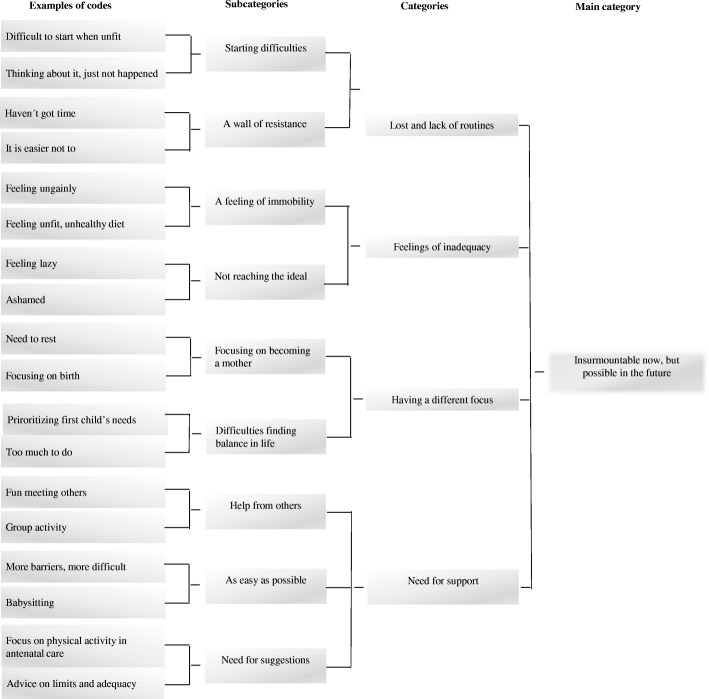
Process of main category construction

The main category “Insurmountable now, but possible in the future” emerged during analysis, and was based on four categories and nine subcategories presented in Fig. 1.

### Insurmountable now, but possible in the future

The women had a positive attitude towards exercise in general, but did not find it possible to exercise during pregnancy, at least not without active support from antenatal care providers or significant others. Some women viewed physical activity as impossible, even with support, while others were more open-minded but without capacity to start on their own. They hoped to start later, as they saw disadvantages in their inactivity and longed for a change in their activity level. They perceived the pregnancy mostly as a barrier to exercise but simultaneously as a motivator and a possibility for better health in the future.

#### Lost and lack of routines

The women all described how they had been exercising more or less earlier in life, at least during their youth while performing different types of leisure time activities and in school during sport lessons. Currently they had no routines for exercise and they expressed several reasons, including pregnancy-related problems and lack of time. These reasons were viewed as barriers that when combined were very difficult to overcome.

### Starting difficulties

The further away their previous routines for exercise were, the larger the barriers had grown. They acknowledged the need for an established routine and motivation, which they lacked. The women with previous positive experience of exercise knew that they had lost a state of well-being and wanted to re-establish this in the future. The women who had not previously felt good or had fun in connection with exercise experienced an additional barrier.

*And in the end I felt that I was very far from exercise and then it’s also more difficult to approach it… And you have to start all over again. And that is tough! And I understand that it will be onerous in the beginning before you get going and really start to think it is fun. And I have never reached that point. (No. 9)*

They found it was easy to postpone their ambitions of exercise, especially during the short period of their life when they were pregnant.

*And then you think that, yes, I will start next week. But now, more than half of the pregnancy has past and that week hasn’t come yet. (No. 11)*

They expressed a hope for a change in the future. These thoughts varied from concrete plans for how and when they would exercise, to a less precise will to start exercising later on. It could also be difficult to find the right activity which would make exercise a more attractive option. If the women had previous sport injuries, they described themselves as active persons, although in some cases it was several years since they had a continuous habit of exercise. They felt sad that they had been forced to stop exercising, but had not yet felt motivated enough to find another type of exercise.

### A wall of resistance

The women described internal as well as external barriers as reasons for not exercising. Internal barriers were connected to lack of motivation, prioritizing other things or choosing the easiest alternative.

*Laziness. Easy-going. It’s like this, now I have worked enough for the day, now I want to go home. I don’t want to get home at six or seven in the evening. I want to come home at half past four, that is reasonable. And then, to go out again when you have come home. That won’t happen! Even if I have thought about doing, no, it won’t happen. (No. 9)*

During the interviews, the women mentioned and returned to their lack of motivation for exercise and the mechanisms that prevented them from establishing routines. They had not explored these issues in depth for themselves. They described that the problem was not that they did not know that exercise was beneficial, but that they did not do it anyway, for reasons that were not always logical or clear to the women.

*I have thought that I ought to exercise, but I haven’t considered it deeper than that, why you choose not to prioritize it or why you don’t have time for it. It becomes some sort of defence mechanism in the head. (No. 13)*

*I do not know. I actually do not know. I really do not know, actually. (No. 1)*

External barriers to exercising were described as the loss of routines, for example, caused by moving from one city to another, including losing friends and established activities. Linked to this was the difficulty of finding a new activity and a feeling of not knowing the existing alternatives, but still they did not seek them actively. If the woman had practised physical activity earlier but had not had a firm routine, it was common that a simple thing had permanent consequences and the routine was broken. For example, getting a cold or missing class could be reason enough for not starting again. Darkness and cold during wintertime were also mentioned as barriers to outdoor activities and a reason for not starting or continuing with, for example, walking or cycling. During pregnancy, the women experienced problems such as nausea, fatigue, and weight gain, which they considered to be major additional barriers.

#### Feelings of inadequacy

The women felt immobile and knew that it would be positive for them if they started to exercise, which they referred to as common knowledge. At the moment, they described a feeling of not reaching their own and society’s ideal in terms of exercise.

### A feeling of immobility

The women felt immobile because of lack of exercise. In addition, they experienced bodily changes due to their pregnancy and they described their condition in terms of being tired without any energy, being ungainly, heavy and stiff. This caused dissatisfaction and they missed the days when they had been more active.

*And I already feel that I am not so supple as last time (during her first pregnancy). And then I feel that I have to do something before giving birth. So it won’t be so tough. Because I already feel ungainly. (No. 1)*

Pelvic girdle pain was expressed by two thirds of the women and this considerably added to the feeling of immobility as it restricted their general mobility as well as their self-perceived possibility to exercise.

The women looked forward to become mobile again, and for those who perceived themselves as overweight, to losing weight, which motivated them to take exercise in the future. Besides lack of exercise, these women also linked their feeling of immobility to their weight gain during pregnancy.

### Not reaching the ideal

All of the women saw the advantages of exercise and described it as an ideal state. It was something they knew was beneficial, but at the moment unreachable.

Not meeting society’s ideal concerning exercise was a component that contributed to a feeling of dissatisfaction.

*I am a little bit ashamed of it. That I can’t do something about it. And that I haven’t done it in the meantime (during pregnancy). And now it’s starting to feel a little bit late. (No. 15)*

The women were positive to everyday activities, such as climbing stairs instead of taking the lift, to somewhat compensate for their inactivity and make them feel less discontent with their own inactivity. One woman described why she walked instead of taking the bus:

*Well, I guess it’s like, in my mind, you know, I can tell myself “I did do some exercise today”. I wasn’t just, you know, like you feel lazy and you feel “I am doing something”. (No. 16)*

Besides the recognition of the physical benefits of activity, the women also expressed a longing for mental satisfaction, such as becoming happier, gaining mental strength and sleeping better at night.

#### Having a different focus

Women described how the pregnancy had changed their focus in life and they concentrated on their pregnancy and struggled to find balance in life.

Focusing on becoming a mother.

The additional strain from pregnancy-related issues such as tiredness partly prevented the women from physical activity and made them prioritize rest during this period of life. Becoming a mother was the primary focus for the women to justify or make it acceptable for themselves to postpone their initiative to start taking regular exercise.

*It feels like I’m more focused on the fact that it is will soon be due, so now I’m going to prepare mentally more than doing that (exercising). I’ll have to start to exercise later instead. (No. 2)*

All women had much faith in the future concerning their ambitions to exercise. They expressed hope that the parental leave would be the opportunity they needed to change their habits. Women who wished to lose weight included healthy diet in their imagined post-pregnancy concept of health. Walking with the stroller was viewed as an attractive and attainable goal.

Later on, other types of activities together with the child were a possible and positive opportunity that the women identified. The women wished to become active not only for their own sake, but also for their children’s. The women explained that it was important for them to live long and healthy and to be able to play with their children.

*… you have to be able to be an active parent and play with them and so on. So I believe it’s better to strengthen the body and be more alert and have more strength. Just generally. If I feel well, they will too. I think so, everybody feels good if I exercise. So that’s the main reason. (No. 14)*

Some women mentioned that they wished to become a role model for the child regarding exercise so that early healthy lifestyle habits could be established for the children.

### Difficulties finding balance in life

The women all portrayed daily life activities both as real barriers and as excuses for not exercising. Family life was highly valued and long working days were unavoidable. The pregnancy made them more tired than usual and the women felt that the days did not have enough hours. Prioritizing themselves had to be put on hold. When all compulsory daily life activities were performed, the women had little or no strength for exercise. It was easier not to be physically active and thereby gain more time for other activities.

*There is a lot to do when you have a house, and stuff like that, when you come home. So then you say “I’ll take it next day” and then maybe you get a cold, and some time passes, and you have to wait until you are well. And then you have a lot of other things to do because you have been ill, so it… there are excuses and that kind of thing too. (No. 5)*

The women prioritized previous children before spare time of their own, but sometimes the children’s needs promoted shared physical activities when playing together or for example when cycling or walking in the forest.

#### Need for support

Initiating exercise after or during pregnancy was perceived as easier with support from others and if the women knew about easily accessible alternatives suitable for pregnant women, such as group activities or easy exercises to perform at home.

### Help from others

There were divergent opinions about the degree to which a significant other person, such as husband or friend, or a group activity was valued. Both the presence of an inspiring instructor and being accompanied by a friend could be motivating. Several of the women referred to their partner as actively trying to encourage them to start exercising.

Some women described how a group of other pregnant women would be beneficial as they would exercise on the same terms. Nevertheless, it was difficult to start on their own if the women classified themselves as untrained, as they imagined it to be uncomfortable when being both pregnant and a beginner among non-pregnant participants or pregnant participants who were used to taking exercise.

*I have read that they will have some kind of yoga for pregnant women. But I feel I am totally unfit. So it feels silly to go there by myself. That’s how I feel. (No. 11)*

While some women solely referred to their own motivation as the crucial factor for starting to exercise, others referred to the organization of Swedish antenatal care, with just a few antenatal visits in early pregnancy. This was pointed out as a problem, if the women wished to be active but were in need of professional support.

*…and that is probably another thing, not having that (antenatal visits) regular… you have a gap from about week 9 or 10 to week 25, when you are just kind of on your own and you feel OK, but you just carry on without doing it… so you don’t have that reminder or kind of pressure. (No. 14)*

### As easy as possible

At the same time as there was a need for routines, it was felt that exercise should ideally be easily accessible as regards both time and location. A long distance to the sport facilities or limited opportunities to choose a suitable time were perceived as barriers by the women.

*The smallest barrier is just to put on trainers and maybe a warm coat, and take a walk. For the more barriers you build, the more difficult are they to overcome. You have to book, rebook and you have to go there, and you have to take a shower, and you have to fix and plan. And then it falls through just from thinking about it. So it has to be, it will probably be walks and running again, for that is the smallest barrier. (No. 7)*

It was suggested that exercise should be more mandatory and not in forms that could be questioned. For example, having a dog or getting a formal physical activity prescription from antenatal care would make it easier as this would be perceived as not having a choice.

*This is an area that has to be prioritized more; that it is incorporated (in the antenatal programme) like a natural part as much as many other parts, so to speak… Because I think it would be, sometimes you should not have the feeling of too much of a free choice. That it’s included. (No. 3)*

Although the women planned to become more physically active after birth, they also recognized that their time would be limited and they would have to prioritize the newborn baby’s needs before their own. It was considered difficult to have too detailed plans in advance, but activities that allowed them to bring their child along was one option identified by the women.

### Need for suggestions

It was not considered necessary that the midwife should be an expert on exercise, but the women would like her to be a catalyst that presented alternatives suitable for pregnant women and could refer them to suitable activities or, for example, to a physiotherapist. Just to have knowledge about organized alternatives in the neighbourhood would have been of value for the women or to know about instructive exercise websites targeting pregnant women. Furthermore, the women called for recommendations for suitable mild forms of activities that could be performed at home and easily incorporated into daily life. It was a common experience that the subject of exercise was only taken up at the beginning of pregnancy and the women wished that exercise should also be discussed during subsequent antenatal visits. Instead it was suggested to be practised or at least followed up as pregnancy gradually influenced the women’s abilities and possibilities to be active. The pregnancy generated additional questions concerning suitable forms of exercise to be performed and how these should be adapted, in case of health problems such as pelvic girdle pain.

*I think that it should be more in focus and that they should discuss it more often. You know, present recommendations and focus more on it at the appointments with the midwife. So you are updated on how it goes, if there are any difficulties with it, like that. And maybe have some information about why it is good for you. (No. 4)*

To practise some kind of exercise in connection with antenatal care or antenatal class and not just to discuss it was one solution mentioned.

Furthermore, as other issues seemed to be more in focus, the women did not consider introducing exercise as a topic themselves and it was therefore important that the subject was initiated by the midwife if the exercise was to be acknowledged during pregnancy for these non-exercising women.

## Discussion

This study explored non-exercising pregnant women’s views and experiences of exercise and found that in general the women had a positive attitude towards exercise. However they did not find it possible to practise during pregnancy – at least not without active support from antenatal care providers or significant others. Some women viewed exercise as impossible, even with support, while others were more open minded but without own capacity to start. They hoped to start later, as they saw disadvantages of their inactivity and longed for a change in their activity level. They perceived the pregnancy mostly as a barrier to exercising but at the same time as a motivator and a possibility for better health in the future.

### Not overcoming barriers

The participants in this study were not comfortable with their level of exercise, but experienced more barriers than resources to mobilize themselves to engage in exercise. The barriers experienced were lack of time and physical pregnancy-related symptoms, as also shown by other researchers [22, 23]. Additionally, this group of non-exercising pregnant women had lost, and therefore lacked, routines for exercise. This generated great initial difficulties which diverges from previous research showing that experiences and already established habits could be helpful in overcoming barriers [24].

### Intentions to exercise

According to the current results, the women were aware of the benefits of exercise but found it difficult to combine with their major focus on becoming a mother. Among the women who had an intention to start exercising during pregnancy, but had not managed it, some had difficulties explaining why. Other studies have shown that many women consider rest to be more important than activity and exercise during pregnancy [29, 30]. The belief that rest is beneficial during pregnancy has been shown to be supported by women’s networks [30], but not shown in the current study, where partners were referred to as potential supporters concerning exercise. Family members’ strong normative influence on women’s exercise during pregnancy and postpartum has been recognized by Symons Downs and Hausenblas [31]. However, other and stronger forces were explained as preventing the women in the current study from exercising. An interesting result of this study was the women’s intention and willingness to start exercising after the birth. ACOG [2] states that it is important to support lifelong health habits during pregnancy and exercise between pregnancies may have positive impact on fitness, mood and weight loss [32]. The latter is important as weight gain between pregnancies increases the risk of adverse pregnancy outcomes including stillbirth [33, 34]. The women described that they wanted to be role models for their children in relation to exercise. This is an important result as there is some evidence that maternal inactivity during pregnancy may contribute to child obesity risk in both active and inactive children 3–9 years old [35].

### Need for support from care givers

The women’s need for support from antenatal health care professionals varied concerning both extent and shape, stressing the need for individualized care. Although some women did not imagine that any support would help them to start exercising during pregnancy, most regretted the lack of *continuous* support for exercise during pregnancy that could be in accordance with their evolving physical changes due to pregnancy. The women even called for elements of mandatory exercise in antenatal care. Interventions during pregnancy, especially face-to-face counselling, have shown some success in reducing the decline of physical activity in pregnant women [36]. A qualitative study with participants having lifestyle discussions in a health centre setting has shown that a desire to make a lifestyle change combined with sensitivity in the discussion are basic conditions for success [37]. Furthermore, this is in line with the experiences of pregnant women participating in group exercise sessions [24].

Group interventions exclusively for pregnant women may be warranted as the women in the current study pointed out that it was important for them in order to dare as beginners and that the exercise programme should be adapted to their pregnancy. Hinton and Olson [18] have shown that self-efficacy is a predictor of change in physical activity. However, a systematic review evaluating the evidence for different interventions aiming at improving physical activity during pregnancy concluded that in general little is known about the efficacy of the interventions [38].

The women’s individual needs of support underline the importance of health professionals following the current recommendations for counselling on exercise. ACOG [2] states that an exercise programme meeting the recommendations should be developed with the woman and adjusted when medically indicated, but the result of our study reflects a lack of such structured support. An interview study with 41 midwives counselling on physical activity, showed that this was a complex task containing both opportunities and challenges, for example difficulties in responding to divergent needs while struggling with lack of resources and fear of failure as expressed by the midwives [39]. To improve counselling, further training for midwives and resources should be introduced [39]. This is especially important for preventing social differences in health, as both previous Swedish and international research has identified social inequality in meeting the recommendations for exercise during pregnancy [5, 18, 21].

To understand why pregnant women do not start exercising, the theory of planned behaviour [40] may be of use. The theory includes the concept of perceived behavioural control which describes a person’s perception of how easy or difficult it is to perform specific behaviours. Perceived behavioural control may include both practical issues, which in the current context for example can be explained as easy access to exercise, and the perception of one’s own abilities. A person’s level of perceived behavioural control, combined with the level of intention (motivation), may in this context potentially determine whether the pregnant woman actually starts to exercise or not. According to Ajzen [40], the level of intention is influenced by a person’s attitude to the behaviour as well as perceived social pressure. Interventions targeting exercise should acknowledge the complexity in a woman’s decisions concerning exercise during pregnancy.

### Limitations

The sample of women in this study was diverse as regards socio-demographic characteristics such as age and level of education as well as parity. Nevertheless the study only covers three antenatal care centres in Sweden and the results should be interpreted in this context. The transferability to women in developing countries with less well-organized health systems is low or not possible. All authors participated in the analysis process by analysing first individually and then comparatively to increase the confirmability. All authors have preunderstanding as midwives and two of the authors, HKH and MB, have in addition preunderstanding from previous research on physical activity among pregnant women, but not among non-exercising women. The authors’ preunderstanding was recognized and discussed in relation to the analysis.

As the midwives who informed about the study also were the ones that had to counsel the women on exercise, this could be a potential source of bias, as it cannot be excluded that this may have resulted in more focus on counselling on exercise from the midwives during the study period which may have affected the women’s experiences. However since the results of the study revealed a need for further support and continuous follow-up during antenatal care, this effect is likely to have been limited.

### Clinical implications

The fact that the women in this study did not have any current routines for exercise led to an increased need for support compared to women practising exercise pre-pregnancy. An easily implementable implication of this study is to present local alternative examples of exercise offered during pregnancy in the antenatal care setting, as the women in this study were in need of suggestions. Ideally exercise in groups could be incorporated as the social element and having fun with other pregnant women is an aspect that could be a strong facilitator for exercise according to the results of the current study.

The results clearly showed that the women liked to start exercising after birth. Postpartum walking with baby stroller was judged as realistic by the women and could be encouraged as a part of the woman’s individual plan or as a part of the postpartum health care.

## Conclusion

The results provide a deeper understanding of non-exercising pregnant women’s situation and the barriers they try to overcome with respect to exercise, including their lack of routines. Health professionals should take into consideration that the pregnancy is viewed both as a barrier preventing exercise and as a motivator for a healthier lifestyle and therefore a possibility for exercise promotion. Exercise should not only be supported at the beginning of the pregnancy but must be emphasized throughout the pregnancy. As the result showed that these women were highly motivated to a lifestyle change post-pregnancy, it may be crucial to support non-exercising women postpartum.

It is essential to conduct further research developing a behaviour-change intervention targeting non-exercising pregnant women, including continuous support during and after pregnancy followed by a RCT.

## Additional file

Additional file 1: Interview guide to the qualitative study “*Not now but later – non-exercising pregnant women’s views and experiences of exercise”* Brief description of the data: Overall question posed to all of the informants and following clarifying questions posed when needed. Demographic questions. (DOCX 12 kb)
