# Supplementary material for: Not now but later – a qualitative study of non-exercising pregnant women’s views and experiences of exercise
Source: BMC Pregnancy Childbirth. 2018 Oct 11;18:399. doi: 10.1186/s12884-018-2035-3 (PMC6180661; doi:10.1186/s12884-018-2035-3)
Supplement: Supplementary file 1 — Interview guide to the qualitative study “Not now but later – non-exercising pregnant women’s views and experiences of exercise” Brief description of the data: Overall question posed to all of the informants and following clarifying questions posed when needed. Demographic questions. (DOCX 12 kb) [file 12884_2018_2035_MOESM1_ESM.docx]

Interview guide

The same overall question were posed to all of the informants; *“What are your views and experiences concerning exercise before and during pregnancy?”* The informants were asked to speak freely.

This was, when necessary, followed by clarifying questions; for example *“What do you mean?”*, *“Can you explain further?”, “In what way?” “What did you think about that?”.*

At the end of the interview, all of the informants were asked for their age, education, parity and occupation.
